# Early-Onset Degenerative Bone Changes as a Manifestation of Alkaptonuria: A Case Report

**DOI:** 10.7759/cureus.96028

**Published:** 2025-11-03

**Authors:** Rui Braga, Fábia Teixeira, Cátia Matos

**Affiliations:** 1 Family Medicine, Unidade Local de Saúde de Gaia e Espinho - Unidade de Saúde Familiar (USF) Espinho, Espinho, PRT

**Keywords:** case report, degenerative joint disease, early-onset osteoarthritis, homogentisic acid (hga), ochronosis, rare genetic disorder, rare metabolic disorder, alkaptonuria

## Abstract

Alkaptonuria is a rare inherited metabolic disorder characterized by progressive pigment deposition in connective tissues, leading to early degenerative joint disease and tendon fragility. Although uncommon, its clinical manifestations may closely resemble more prevalent musculoskeletal conditions, often resulting in delayed diagnosis. We present the case of a 53-year-old woman with long-standing, disabling osteoarticular complaints initially attributed to common degenerative disease, but markedly premature for her age. Due to worsening bilateral gonarthrosis, she underwent left total knee arthroplasty, during which intraoperative black discoloration of cartilage was observed, consistent with ochronosis. Physical examination revealed bluish pigmentation of the sclerae, auricular cartilage, and tympanic membranes, notably asymmetric, as well as urine that darkened after air exposure. Histology confirmed ochronotic pigment with severe cartilage degeneration and chronic synovitis. Gas chromatography-mass spectrometry demonstrated markedly elevated urinary homogentisic acid (7,016 µmol/mmol creatinine), confirming the diagnosis. Additional findings included lumbar disc disease, osteoporosis, and a non-obstructive renal calculus, although a causal relationship with alkaptonuria could not be established. She later sustained a low-energy intertrochanteric fracture, surgically managed. This case reinforces how common musculoskeletal presentations may mask rare metabolic disorders, emphasizing the importance of recognizing subtle pigmentary clues, including asymmetric otologic involvement, to avoid diagnostic delay through timely collaboration between primary and hospital care.

## Introduction

Alkaptonuria is a rare autosomal recessive metabolic disorder caused by mutations in the HGD gene, which encodes the enzyme homogentisate 1,2-dioxygenase (HGD). Deficiency of this enzyme leads to the accumulation of homogentisic acid (HGA), which is partially excreted in the urine and progressively deposited in tissues, reaching concentrations up to 2,000 times higher than normal [1].

Chronic HGA deposition results in a dark pigment resembling melanin, termed ochronosis, which accumulates in cartilage, ligaments, tendons, bones, cardiac valves, sclerae, skin, and other organs [2,3]. Its deposition weakens connective tissue, increasing susceptibility to injury and degeneration [4,5].

Alkaptonuria is characterized by dark-colored urine, arthritis of large joints, and ochronosis (bluish to black pigmentation) of cartilage and collagenous tissues. This pigmentation manifests as articular ochronosis with early and progressive degenerative arthritis, often involving the spine, knees, hips, and shoulders [6].

The clinical presentation may mimic common osteoarthritis or rheumatologic diseases, contributing to diagnostic delay [7,8], and is frequently established only after the intraoperative identification of characteristic findings during orthopedic procedures [9,10]. Diagnosis is based on the detection of urinary HGA, which can be confirmed through laboratory methods and clinical assessment [11].

With an estimated prevalence ranging from one in 100,000 to one in 250,000 individuals worldwide, rising to approximately one in 19,000 in certain endemic regions such as Slovakia, the condition often remains underdiagnosed or recognized late due to its rarity and non-specific clinical features [12]. Although it does not significantly reduce life expectancy, it is associated with functional limitations and a negative impact on quality of life [13].

Alkaptonuria is a chronic disorder for which no curative treatment currently exists, and management is primarily symptomatic. Nitisinone, a potent inhibitor of 4-hydroxyphenylpyruvate dioxygenase (HPPD), reduces HGA production and represents a promising therapeutic approach when combined with a low-protein diet and supportive measures such as pain control, physiotherapy, and orthopedic surgery [14].

The description of clinical cases is essential to raise awareness among the medical community for the early diagnosis of this rare condition. We present the case of a 53-year-old woman with long-standing osteoarticular complaints, whose investigation of early-onset bone degenerative changes led to the diagnosis of alkaptonuria.

## Case presentation

A 53-year-old Caucasian woman, born in France and residing in Portugal, is divorced and lives in a single-parent household with her 19-year-old daughter. She completed secondary education (12 years of schooling) and works as an administrative assistant.

Her past medical history included dyslipidemia, hypothyroidism associated with Hashimoto’s thyroiditis, and non-obstructive nephrolithiasis. Surgical history included left tympanoplasty for tympanic membrane perforation in 1999 and left total knee arthroplasty in 2025. Obstetric history was G2P1, with one spontaneous abortion and one living child. She was an active smoker (seven pack-years) and denied alcohol consumption.

At around 30 years of age, she developed persistent, mechanically induced arthralgia with progressive worsening. Symptoms initially affected the knees and later extended to the lumbar spine, shoulder girdle, and ankles, progressively leading to significant functional impairment, particularly after the age of 45, resulting in gait limitation and daily need for analgesic therapy.

Physical findings included lumbar hyperlordosis with L4-L5 and L5-S1 discopathy, diagnosed in 2011. In 2016, she declined referral to an orthopedic spine consultation, expressing no motivation for surgical treatment. She also presented with bilateral tricompartmental gonarthrosis, for which total knee replacement was proposed in 2020 but declined, as well as bilateral shoulder osteoarthritis. Tendinosis of the Achilles tendon was also noted.

She was evaluated in Rheumatology for polyarthralgia and degenerative changes considered premature for her age and underwent rheumatologic workup including rheumatoid factor, anti-cyclic citrullinated peptide (CCP) antibodies, antinuclear antibody (ANA) profile, and inflammatory markers, all of which were negative or within normal limits, leading to discharge without identification of a specific rheumatologic disorder.

**Figure 1:**
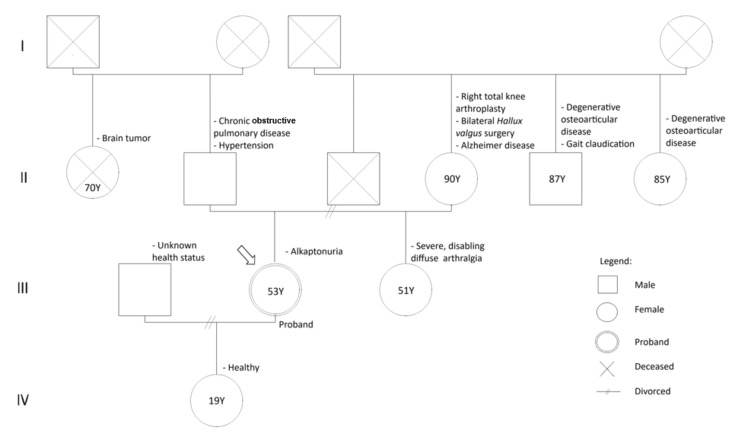
Family genogram of the proband with alkaptonuria The proband (double-outlined circle) presented with disabling diffuse arthralgia and a confirmed diagnosis of alkaptonuria. The patient’s mother and siblings exhibited degenerative osteoarticular disease of varying severity

Regarding family history, several members reported osteoarticular complaints. Her mother had undergone right total knee arthroplasty and bilateral hallux valgus surgery, suggesting significant degenerative joint disease. Her sister, currently aged 51, experiences disabling diffuse arthralgia, without a confirmed diagnosis or clinical stigmata of alkaptonuria to date. There were no other relevant familial conditions reported. Thus, although no previously established cases exist within the family, the presence of this disease in other relatives cannot yet be excluded. The patient’s family genogram is shown in Figure 1.

In 2023, there was a worsening of bilateral knee pain, resulting in significant functional limitation. This condition coincided with the loss of her job and the onset of depressive symptoms, which she associated with chronic pain and disability, leading to the initiation of antidepressant therapy.

Over the years, she underwent multiple radiographs of the knees, which consistently showed progressive tricompartmental degenerative changes disproportionate to her age, as well as bilateral glenohumeral osteoarthritis. She completed several cycles of physical rehabilitation targeting the knees, shoulders, hips, and spine, with only transient symptom relief. Despite optimized conservative management, including physical therapy, analgesics, and activity modification, functional decline continued to worsen. By June 2024, she was severely functionally impaired, being able to walk only with the aid of two crutches, and reported having no quality of life. She was again referred to Orthopedics, where total knee arthroplasty was proposed and, this time, accepted. During surgery, areas of the knee joint with black discoloration were observed, a characteristic finding of ochronosis, which prompted further investigation and referral to a Metabolic Diseases consultation.

**Figure 2:**
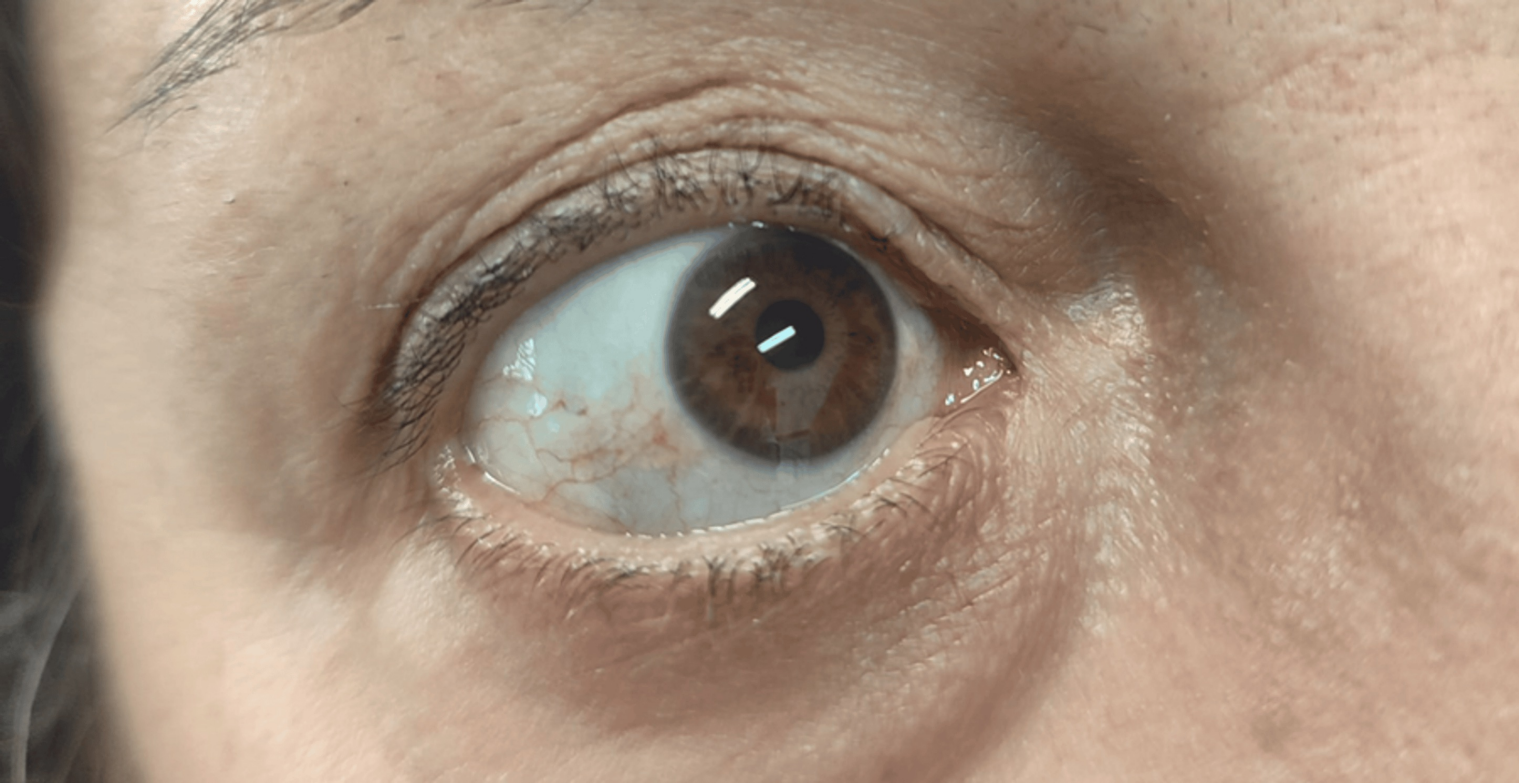
Sclerae with bluish discoloration, a characteristic finding of ochronotic pigment deposition

**Figure 3:**
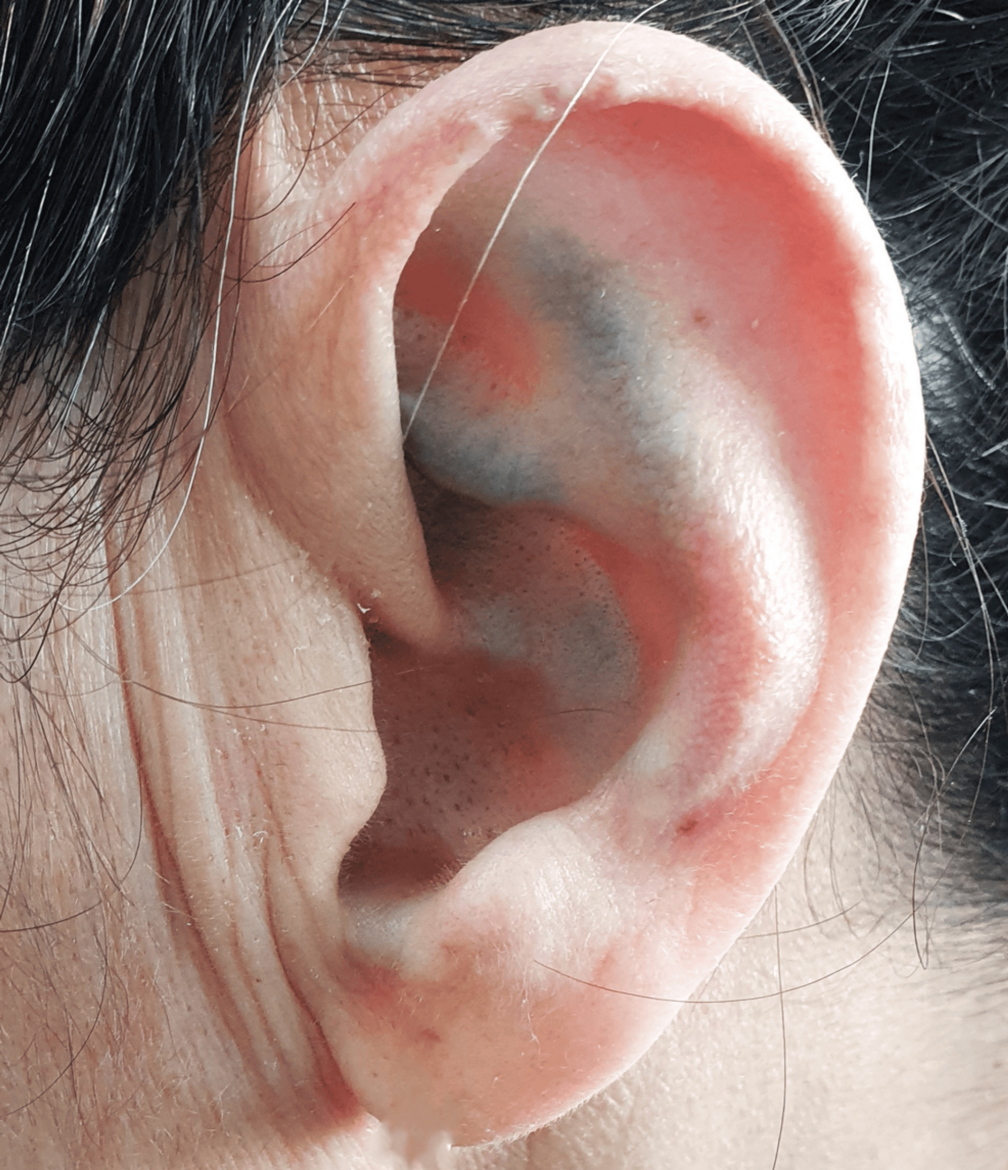
Auricular cartilage with bluish discoloration, suggestive of ochronotic pigmentation

**Figure 4:**
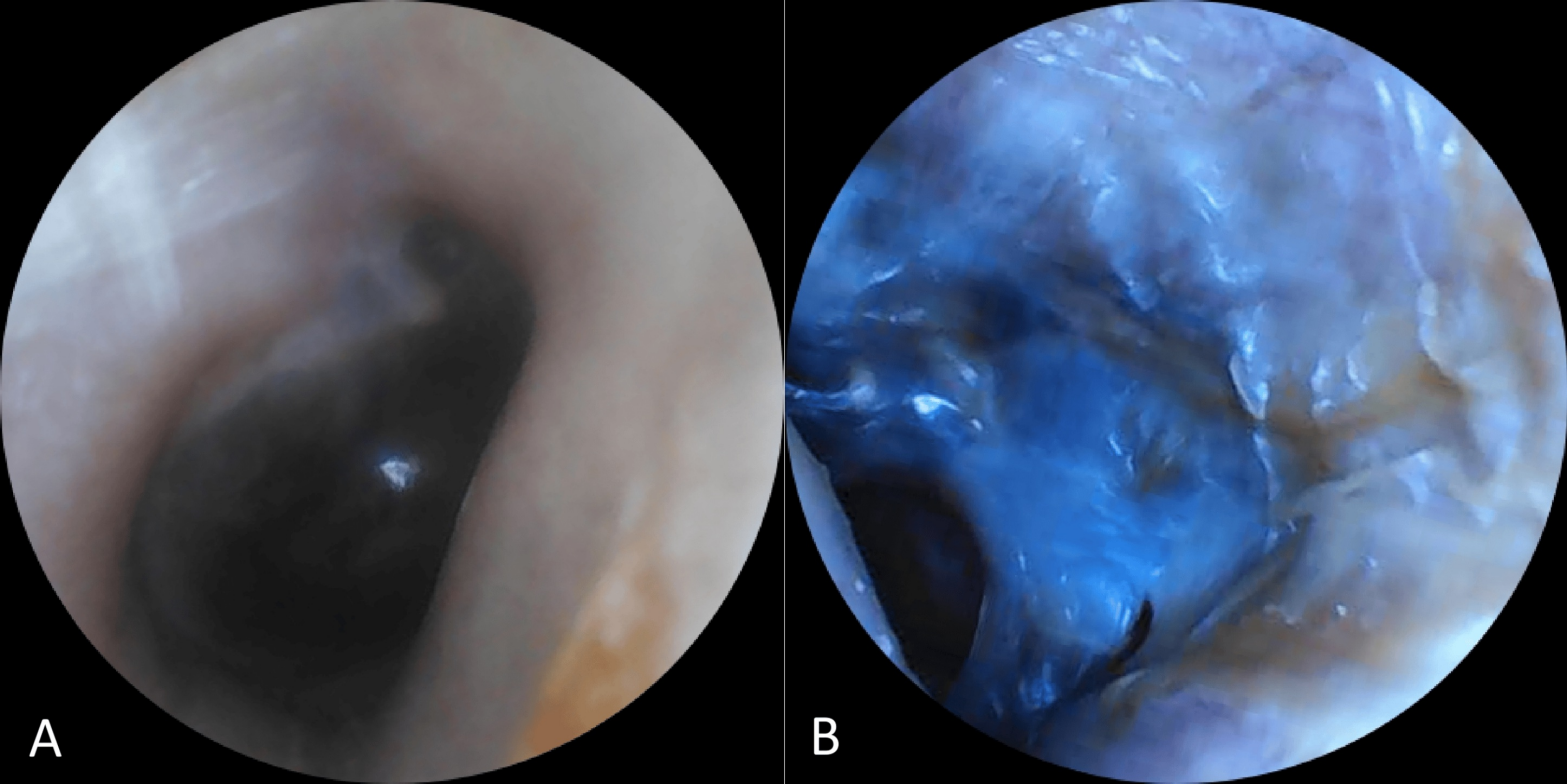
Otoscopic view demonstrating marked asymmetry in tympanic membrane appearance (A) Right tympanic membrane with normal coloration; (B) left tympanic membrane showing diffuse bluish discoloration, consistent with ochronotic pigment deposition

**Figure 5:**
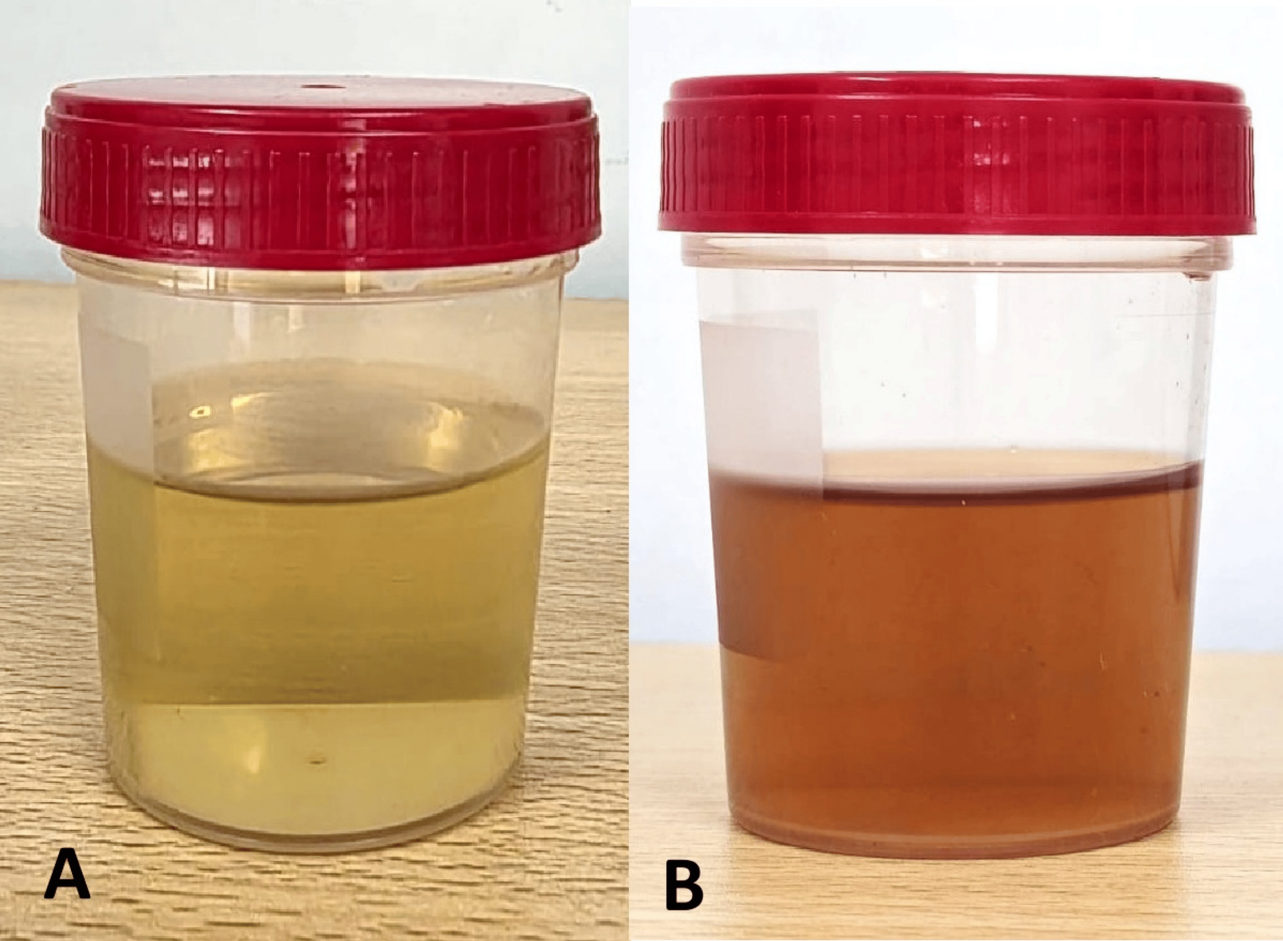
(A) Freshly voided urine sample; (B) urine after 24 hours of air exposure, showing progressive darkening due to homogentisic acid oxidation

Histopathological examination of the joint specimens revealed bone tissue with black pigmentation in the tibial plateaus and other fragments, as well as hyaline cartilage showing marked degenerative changes, including fissures, fragmentation, and clusters of chondrocytes, associated with chronic synovitis. Already aware of the condition, the patient reported to her family physician that she had noticed bluish discoloration of the ears, first observed around the age of 25 and progressively increasing over time, but never previously considered clinically relevant. She also recalled that, during childhood, her urine had a dark color on diapers and bedsheets, which had likewise gone unappreciated at the time. On physical examination, bluish pigmentation of the sclerae, tympanic membranes, and auricular cartilage was confirmed, as well as urine darkening after 24 hours of exposure to air (Figures 2-5 - patient photographs).

Given the clinical suspicion of alkaptonuria, complementary investigations were performed to screen for potential complications. Transthoracic echocardiography revealed no valvular thickening, regurgitation, or aortic root dilation, ruling out early ochronotic cardiac involvement. Renal and bladder ultrasound revealed a non-obstructive calyceal calculus (4 mm) in the upper pole of the left kidney, without hydronephrosis.

Bone densitometry demonstrated bone mineral density values of the lumbar spine at 1.28 g/cm² (Z-score 1.2), femoral neck at 0.658 g/cm² (Z-score -2.0), and total proximal femur at 0.571 g/cm² (Z-score -3.2), which, in correlation with clinical findings, were consistent with osteoporosis. Laboratory evaluation revealed normal serum calcium (8.7 mg/dL), phosphate (3.5 mg/dL), alkaline phosphatase (83 U/L), vitamin D (89 nmol/L), and parathyroid hormone (33.8 pg/mL), ruling out secondary metabolic bone disease. She reported regular menstrual cycles and no history of premature ovarian insufficiency or amenorrhea and was not yet menopausal.

In August 2025, she sustained a left intertrochanteric femoral fracture following a low-energy fall from standing height at home and underwent surgical fixation, after which she initiated postoperative physical rehabilitation. Shortly thereafter, in September 2025, urinary organic acid analysis by gas chromatography-mass spectrometry (GC-MS) was performed at the National Institute of Health Dr. Ricardo Jorge (Portugal) on a urine sample, demonstrating a markedly elevated HGA excretion of 7,016 µmol/mmol creatinine (reference value: undetectable). These biochemical findings confirmed the accumulation of this metabolite, which is pathognomonic of alkaptonuria. Laboratory analysis also revealed an elevated erythrocyte sedimentation rate (45 mm/h), with complete blood count, liver function tests, thyroid function, C-reactive protein, and serum protein electrophoresis within normal ranges. Molecular analysis of the HGD gene is currently in progress to identify pathogenic variants associated with this enzymatic deficiency.

The timeline summarizing the patient’s clinical evolution is presented in Table 1.

**Table 1:** Timeline: clinical evolution of the patient from childhood to the definitive diagnosis of alkaptonuria

Date (age)	Relevant clinical event	Key diagnostic findings	Intervention/outcome
Childhood	Dark urine in diapers, not valued	No investigation performed	No intervention
25 years	Appearance of bluish discoloration of the ears, not valued	No investigation performed	No intervention
30 years	Onset of persistent arthralgia, predominantly in the knees	Early degenerative changes on radiographs	Conservative management (analgesics, physiotherapy)
2016 (44 years)	Orthopedic consultation for lumbar pain	L4–L5 and L5–S1 discopathy	Declined spinal surgery. Conservative management (analgesics, physiotherapy)
2020 (48 years)	Orthopedic consultation for progressive knee pain	Radiographs: bilateral tricompartmental knee osteoarthritis	Declined total knee arthroplasty
May 2024 (52 years)	Rheumatology evaluation for polyarthralgia	Autoimmune and rheumatologic workup negative	Discharged without a specific diagnosis
June 2024	Progressive gait limitation, dependent on two crutches	Severe functional impairment reported. Conservative measures no longer effective	Referred again to Orthopedic Surgery consultation
March 26, 2025	Total left knee arthroplasty	Intraoperative ochronotic pigmentation observed	Surgery performed successfully
April 22, 2025	Family Medicine consultation	Recognition of auricular, scleral, tympanic pigmentation and dark urine	High clinical suspicion of alkaptonuria established
August 4, 2025	Left intertrochanteric fracture after low-impact fall, surgically treated	X-ray confirming intertrochanteric fracture	Surgical fixation followed by initiation of postoperative physical rehabilitation
September 30, 2025	Consultation in Metabolic Diseases	Markedly elevated urinary homogentisic acid. Genetic testing initiated	Biochemical confirmation of alkaptonuria

Following confirmation of the diagnosis, treatment with nitisinone, currently approved by the European Medicines Agency for alkaptonuria, was discussed. Potential benefits, limitations, and adverse effects were explained, and formal authorization through the national regulatory pathway (CFT) is currently being considered. In parallel, given the favorable outcome of the previous left total knee arthroplasty, she has already been proposed for right total knee replacement, which she is currently considering.

The patient expressed frustration with the progressive functional impact of pain and gait limitation, particularly due to the delay in obtaining a definitive diagnosis. Following confirmation of alkaptonuria, she reported relief in finally having a clear explanation for her long-standing symptoms, while acknowledging concern regarding future complications. She expressed willingness to share her case in order to promote earlier recognition and appropriate referral in similar patients.

## Discussion

Alkaptonuria is a rare metabolic disorder whose diagnosis is often delayed due to the non-specific nature of its initial manifestations and their similarity to common osteoarticular conditions. The present case illustrates this reality, with diffuse, disabling, and long-standing osteoarticular symptoms managed by multiple specialties without a definitive diagnosis being reached.

The presentation with disproportionately early-onset osteoarthritis and auricular/ocular pigmentation helps distinguish this disease from primary osteoarthritis. The absence of typical inflammatory or axial criteria, along with markedly elevated urinary HGA, rules out spondyloarthropathies, supporting the diagnosis of ochronotic arthropathy.

Although ochronotic deposition is classically systemic and bilateral, isolated reports have described asymmetric otologic involvement, and it has been suggested that local biomechanical factors may influence the distribution and severity of pigment accumulation. Interestingly, our patient presented with marked unilateral ochronotic discoloration of the left tympanic membrane, while the right membrane appeared entirely normal. This asymmetry may be partially explained by the patient’s history of recurrent otitis media in childhood that eventually led to tympanic membrane perforation and left-sided tympanoplasty, as ochronotic pigment deposition is known to occur preferentially in previously damaged or surgically altered tissues. Post-mortem and histological studies have demonstrated that ochronosis is not uniformly distributed, but rather tends to develop in areas of mechanical stress, tissue injury, or microinflammation, where collagen integrity is altered and, therefore, more susceptible to pigment binding. This mechanism aligns with the concept described in the literature that “damaged” tissue undergoes pigmentation while “undamaged” tissue may remain resistant, despite equal systemic exposure to HGA [1]. This finding reinforces the importance of careful bilateral otoscopic examination in patients with suspected alkaptonuria, even when only subtle or asymmetric pigmentary changes are present. Although ochronosis is classically described as a bilateral and symmetrical phenomenon, this case adds to the limited number of reports documenting clinically relevant lateral asymmetry, plausibly associated with prior local tissue injury.

The intraoperative discovery of ochronosis, later confirmed by elevated urinary HGA levels, was pivotal to the diagnosis. This finding reflects a phenomenon described in several studies, in which a significant proportion of patients are diagnosed only during arthroplasty or other orthopedic procedures. This highlights the challenge of recognizing typical clinical signs-such as bluish ear pigmentation-often overlooked by both patients and healthcare professionals, contributing to substantial diagnostic delays.

Dark urine, resulting from the oxidation of HGA upon exposure to air, is a classic hallmark of the disease [15]. Alkaptonuria results from a deficiency of the enzyme HGD, leading to a block in the phenylalanine-tyrosine degradation pathway and subsequent accumulation of HGA. Demonstration of markedly elevated urinary HGA concentrations by GC-MS provides diagnostic confirmation, as this metabolite is normally undetectable in healthy individuals.

Renal involvement in alkaptonuria has been described, with an increased risk of nephrolithiasis due to high urinary HGA excretion, reported in up to 28% of patients. In male patients, prostatolithiasis has also been documented as a possible manifestation of ochronotic pigment deposition. These stones may appear dark and exhibit variable compositions, including calcium oxalate and phosphate, sometimes containing ochronotic material [16]. However, in the present case, the presence of a non-obstructive renal calculus cannot be unequivocally attributed to the disease, as compositional analysis was not performed. Nonetheless, this finding reinforces the importance of urinary tract surveillance in patients with alkaptonuria due to the documented risk of renal complications. In addition, an elevated erythrocyte sedimentation rate, despite otherwise normal inflammatory laboratory parameters, may reflect a chronic low-grade inflammatory state associated with progressive deposition of ochronotic pigment in tissues. Furthermore, osteoporosis is also a frequent manifestation, often linked to fragility fractures and likely related to HGA deposition in bone, resulting in alterations of the bone matrix [17].

From a therapeutic perspective, nitisinone, combined with a low-protein diet and supportive measures, represents the main therapeutic approach to alkaptonuria. This drug, still considered emerging and not yet approved for this indication, acts as a competitive and reversible inhibitor of the enzyme HPPD, thereby reducing HGA production and preventing or delaying disease-related complications.

Clinical trials have demonstrated a consistent biochemical effect, with reductions exceeding 94% in urinary HGA excretion [18]. However, this decrease has not always translated into significant clinical improvement, which may reflect the slow progression of the disease, suboptimal dosing of nitisinone, or inadequate selection of outcome measures [19]. More recent evidence indicates that administration of 10 mg daily of nitisinone is well tolerated and effective, not only in reducing urinary HGA but also in attenuating ochronosis and improving clinical signs, suggesting slower disease progression. Despite its biochemical efficacy, careful monitoring is required due to the risk of hypertyrosinemia, which can lead to complications such as keratopathy and other adverse effects [20]. In the present case, nitisinone is currently under formal evaluation as a potential disease-modifying therapy.

In this case, the delayed diagnosis did not prevent advanced osteoarticular damage but enabled surveillance for potential renal, cardiac, and endocrine complications, as well as access to social and supportive care. Diagnostic delay is frequently associated with functional decline and significant psychosocial impact, as illustrated by the patient’s chronic pain and osteoarticular limitations, which affected not only physical autonomy but also emotional well-being and professional activity.

## Conclusions

Due to its rarity and non-specific presentation, alkaptonuria remains a diagnostic challenge that requires a high index of clinical suspicion, particularly in the presence of early-onset or atypical osteoarticular complaints. Recognizing subtle signs, such as pigmentary changes in the auricular cartilage and sclerae or even asymmetric tympanic membrane discoloration, may be crucial to shortening the time to diagnosis and preventing unnecessary investigations. This case underscores the pivotal role of the family physician, not only in ensuring continuity and coordination of care but also in identifying subtle findings that may otherwise be overlooked by patients or specialists. Few cases have been reported in Portugal and Southern Europe, emphasizing the importance of sharing clinical experiences to raise awareness within the medical community. Broader dissemination of such cases may promote earlier diagnosis, improve access to emerging disease-modifying therapies such as nitisinone, and ultimately enhance patient outcomes.
